# Correlation between Mechanical and Morphological Properties of Polyphenol-Laden Xanthan Gum/Poly(vinyl alcohol) Composite Cryogels

**DOI:** 10.3390/gels9040281

**Published:** 2023-03-29

**Authors:** Irina Elena Raschip, Raluca Nicoleta Darie-Nita, Nicusor Fifere, Gabriela-Elena Hitruc, Maria Valentina Dinu

**Affiliations:** “Petru Poni” Institute of Macromolecular Chemistry, Grigore Ghica Voda Alley 41A, 700487 Iasi, Romaniavdinu@icmpp.ro (M.V.D.)

**Keywords:** composite hydrogels, cryogelation, morphology, poly(vinyl alcohol), rheology, uniaxial compression, xanthan

## Abstract

This study aimed to evaluate the effect of the synthesis parameters and the incorporation of natural polyphenolic extract within hydrogel networks on the mechanical and morphological properties of physically cross-linked xanthan gum/poly(vinyl alcohol) (XG/PVA) composite hydrogels prepared by multiple cryo-structuration steps. In this context, the toughness, compressive strength, and viscoelasticity of polyphenol-loaded XG/PVA composite hydrogels in comparison with those of the neat polymer networks were investigated by uniaxial compression tests and steady and oscillatory measurements under small deformation conditions. The swelling behavior, the contact angle values, and the morphological features revealed by SEM and AFM analyses were well correlated with the uniaxial compression and rheological results. The compressive tests revealed an enhancement of the network rigidity by increasing the number of cryogenic cycles. On the other hand, tough and flexible polyphenol-loaded composite films were obtained for a weight ratio between XG and PVA of 1:1 and 10 *v*/*v*% polyphenol. The gel behavior was confirmed for all composite hydrogels, as the elastic modulus (G′) was significantly greater than the viscous modulus (G″) for the entire frequency range.

## 1. Introduction

Polysaccharides such as chitosan (CS), xanthan gum (XG), pullulan, cellulose, alginate (ALG), and dextran meet environmental concerns and appear as promising compounds for the preparation of eco-friendly materials since they are nontoxic, abundant, biocompatible, and biodegradable polymers [1,2]. However, some drawbacks in terms of performance, such as thermal, moisture barrier, and mechanical properties, limit the polysaccharides application domain. To overcome these disadvantages and enhance the physicochemical properties and functional features of polysaccharides, several effective strategies, including cross-linking, blending with other natural or synthetic polymers [3,4,5,6,7,8,9,10,11,12,13,14,15,16,17,18,19,20], or incorporation of antioxidant agents, e.g., phenolic compounds [13,14,15,21,22,23,24,25,26] have been proposed. For instance, blending of CS with natural or synthetic polymers, including xylan [24], starch [25], poly(vinylalcohol) (PVA) [26], or polyethylene terephthalate [27], has been reported to improve the mechanical properties of the composite films. Adding xylan significantly increased the percentage of elongation at break of the composite films, and samples with 20 to 25 wt.% xylan exhibited the highest tensile strength and young modulus values [24]. The combination of two biopolymers has also been pointed out as a nice approach to enhancing the Alg-based films features. In this case, the materials have been synthesized using gelatin and sodium Alg to improve their weaknesses, brittleness, and tensile strength properties [28,29,30].

The mechanical stability of polysaccharide-containing films has also been reported to be enhanced by the embedding of polyphenolic-rich extracts [31,32,33,34,35]. Thus, pliable and flexible bioactive pullulan-based films have been obtained only by adding 1% of extracts from chestnut spiny burs or roasted hazelnut skins [31]. Curcumin-loaded ALG/polyethylene oxide electrospun nanofibers cross-linked with trifluoroacetic acid with reinforced mechanical features have also been described [30]. In another study, the incorporation of 0.2–0.6% of rutin within CS/PVA films significantly enhanced their stiffness and flexibility [32]. The embedment of essential oils into CS/dextrin cryogel matrices generated porous films with increased elasticity, their elastic modulus being 40 times higher than that of pristine films [33]. The enhancement of mechanical performances of these films has been associated with the phase compatibility achieved by strong intermolecular interactions between functional groups of the film components through the formation of hydrogen bonds [30,33]. However, other studies showed a significant decrease of the tensile strength and elastic modulus values when black chokeberry extracts were entrapped within ALG films [34] or cinnamon leaf oils into the CS-based formulations [35].

The impact of the non-covalent complexes formed between polysaccharides and polyphenols on the physical properties of the gels has been described only in a few studies [36,37,38,39,40,41]. It was shown that the addition of phenolic compounds induced the aggregation of polysaccharides in solution as a function of polysaccharide concentration [36]. In another study, the effect of polysaccharide branching degree on arabinan-rich pectic-polyphenol interactions was also proved [37]. It was found that the sugar beet arabinan-procyanidin interactions were more selective toward linear polysaccharides than for the branched ones [37]. The influence of the environmental parameters, including pH, ionic strength, and temperature, on the tannic acid/beta-glucan (TA/BG) interactions has also been investigated [38]. Stable and homogeneous TA/BG complexes were obtained at pH 5, whereas at lower pH values the aggregation and sedimentation was observed. By raising the ionic strength and decreasing the temperature, an increase in the turbidity of the TA/BG complexes was observed, which indicates the formation of hydrogen bonds as well as electrostatic and hydrophobic interactions [38].

On the other hand, rheological investigations are very important tools in the characterization of hydrogel-based materials since they provide valuable information on the viscoelastic features of the gels. For instance, it was found that the viscoelastic properties of ionic semi-interpenetrating polymer networks containing dextran and poly(acrylamide-*co*-*N*,*N*′-methylene(bis)acrylamide) have been influenced by the gel formation temperature, cross-linker ratio, initial monomer concentration, and molar mass of dextran [42]. The incorporation of high molar mass dextran enhanced the mechanical properties of composite hydrogels, while the improvement of hydrogels elasticity was reached by performing the synthesis of hydrogels below the freezing point of the reaction solutions [42]. When evaluating the viscoelastic properties of gels based on XG cross-linked with aluminum lactate, Amaral et al. [43] demonstrated a direct influence of pH, salinity, and temperature on the initial strength of the gel. Strong gels have been obtained at pH 8, 70 °C, and a salinity of 29.94 mg L^−1^ total dissolved solids [43]. Shear thinning, non-Newtonian behavior, and enhanced elasticity have also been demonstrated for terpolymeric hydrogels consisting of XG, poly(acrylic acid), and poly(N-isopropyl acrylamide) by applying some rheological models such as Bingham, modified Bingham, and Ostwald power laws [44]. A complex study evaluated the rheological and texture properties of different types of hydrogels comprised of neutral (hydroxy/alkylcelluloses), anionic (carboxyalkylcellulose and its sodium salt, tragacanth, carrageenan, XG), or cationic (CS) polysaccharides designed for drug delivery applications [45]. It was concluded that the in vitro release of the encapsulated drug significantly varied due to the different viscoelastic properties and to the contribution of the interactions occurring within the polysaccharide-based hydrogels [45].

Lastly, the mechanical properties of composite films are generally influenced by the nature and ratio between components, the initial concentration of monomer or polymer, the cross-linking type (physical or chemical), the degree of cross-linking, the molar mass of components, or synthesis conditions. Recently, in our group, physically cross-linked XG/PVA hydrogel films embedding polyphenolic extracts derived from various types of red grape pomace (Feteasca Neagra or Merlot) and exhibiting improved antioxidant and antimicrobial properties have been developed [13,14,15]. Moreover, the bioactivity of polyphenols depends to a great extent on their content and chemical structure, whereas the effect of various physical processes including heating, drying, irradiation, roasting, ultrasound processing, high-pressure treatment, and the interaction with the polymeric matrices either by non-covalent or covalent bonds determines their structural changes [46,47,48,49]. For instance, the increase of flavonoid content in tomato fruit cuticles during ripening has been correlated with more rigid cutin networks that reinforced the mechanical function of polysaccharides [46]. The addition of *Polygonatum cyrtonema* extracts diminished the light transmittance but improved the UV blocking performance, mechanical properties, and antioxidant activity of carboxymethyl cellulose/XG/flaxseed gum films [49]. To better understand the structure-property relationship of the XG/PVA cryogel films containing polyphenolic extracts of Feteasca Neagra (trans-resveratrol as the main compound identified by HPLC), in this study we have examined their compressive and viscoelastic properties. Moreover, the investigation of the mechanical properties is compulsory since it foresees the composite hydrogel films performance under various compression and shear stress conditions.

## 2. Results and Discussion

In this study, we investigated the mechanical and morphological properties of polyphenol-laden XG composite hydrogels, which were prepared by multiple cryogenic cycles at −20 °C coupled with freeze-drying, according to a procedure previously reported [13]. Briefly, 1 wt.% polymeric solutions were first separately prepared, as follows: the XG aqueous solution was made at room temperature (RT), while the PVA was dissolved in water at 80 °C and cooled to RT before use. Then, portions of each polymeric solution were mixed in various ratios, as presented in Table 1. A homogenous mixture containing both polymers was formed after 24 h of mild stirring at RT. The polyphenolic extract was drop-wise added to the polymeric mixture, and then the whole system was repeatedly frozen and thawed for cycles III to VII. Finally, all samples were dried by lyophilization or solvent casting. Some optical pictures with the obtained materials are presented in Figure S1, Supporting Information. The 3D cryogenically-structured networks of XG-based composites were fabricated through hydrogen bonding formed by blending XG, PVA, and polyphenolic extract derived from Feteasca Neagra type red grape pomace. The sample codes, their compositions, and some characteristics of XG-based composite hydrogels are listed in Table 1. The materials preparation was repeated three times for each parameter setting. Each sample code consists of “CG” from the composite gel followed by a number 100, 90, 75, or 50, which represents the fraction of XG in the sample, then “F” from the variety of the grape pomace extract, i.e., Feteasca, and finally the Roman numbers III, V, or VII, which indicate the number of freeze-thaw cycles used for the synthesis of the composite hydrogels.

The evaluation of swelling ratio, contact angle, and pore sizes of polyphenol-laden XG/PVA composite hydrogels in comparison with neat hydrogel networks comprised of only XG or PVA (Table 1) pointed out the great influence of the synthesis conditions on these parameters. Thus, by increasing the number of freeze-thaw cycles, the water retention capacity, i.e., swelling ratio, drastically decreased, indicating a strong interaction between the functional groups of XG, PVA, and polyphenolic extracts. The surface wettability of all composite hydrogel films was studied by measuring the contact angle (θ°). The increase in θ° with the rise of PVA content and number of freeze/thaw cycles to seven (Table 1) indicates the increase in hydrophobicity of all hydrogel films, which further supports the successful embedding and great stability of polyphenolic extracts within polymeric matrix. Changes in the mechanical and morphological features of these composite hydrogels were analyzed in the next sections in terms of the number of cryogenic cycles, the ratio between polymeric components, and the presence of polyphenolic extract within 3D hydrogel networks.

### 2.1. Compressive Behavior

**Figure 1:**
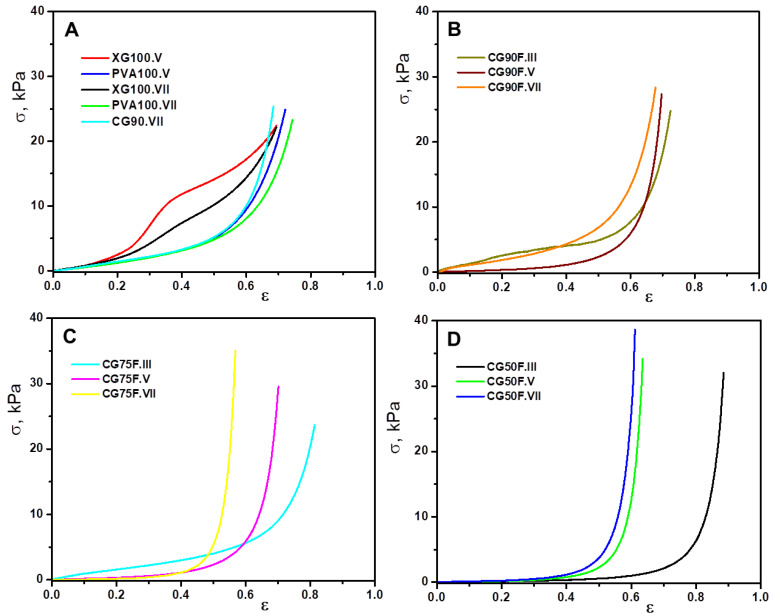
Stress-strain profiles of lyophilized XG-based composite hydrogels obtained by uniaxial compression using a cross-head speed of 0.2 mm min^−1^ and a force of 20 N: (**A**) control samples; (**B**) composite hydrogels prepared with a weight ratio between XG and PVA of 9:1; (**C**) composite hydrogels prepared with a weight ratio between XG and PVA of 3:1; (**D**) composite hydrogels prepared with a weight ratio between XG and PVA of 1:1.

The elasticity, toughness and stability of the polyphenol-loaded XG/PVA composite hydrogels were evaluated by uniaxial compressive measurements. We were interested in investigating the influence of the ratio between polymeric components, the number of cryogenic cycles, and the presence of bioactive compounds on the compressive mechanical performance of the XG-based hydrogels. All composite networks exhibited typical compressive stress-strain (σ-ε) profiles characteristic of materials prepared by cryogelation (Figure 1). Thus, all samples either containing polyphenol or not can be compressed to over 50% strain, without any fracture development, which is attributed to their macroporous structures.

**Figure 2:**
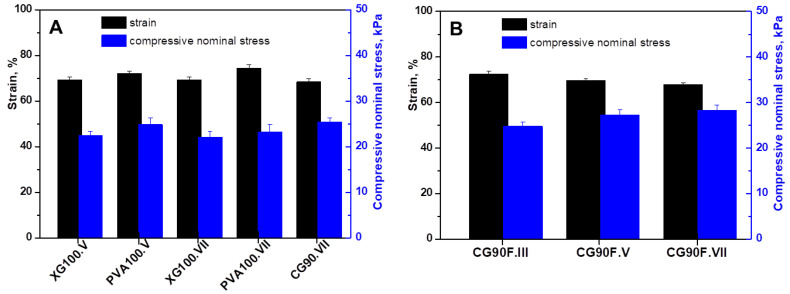

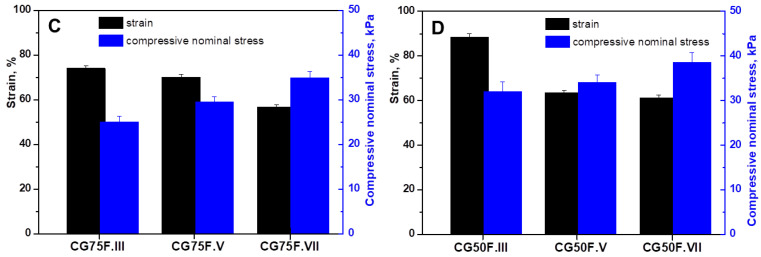
Maximum sustained compression (black color) and compressive strength (blue color): (**A**) control samples; (**B**) composite hydrogels prepared with a weight ratio between XG and PVA of 9:1; (**C**) composite hydrogels prepared with a weight ratio between XG and PVA of 3:1; (**D**) composite hydrogels prepared with a weight ratio between XG and PVA of 1:1. All the data were expressed as the mean of three individual measurements ± SD. The SD is presented as error bars.

The values of the mechanical parameters, namely the maximum sustained compression and the compressive nominal stress of the polyphenol-loaded XG/PVA composite hydrogels obtained from the stress-strain curves, are represented in Figure 2.

Compression measurements performed on composite hydrogels with different XG/PVA composition ratios and three cryogenic cycles showed common behaviors, but with some features that can be associated with the network composition and cryogenic parameters. From the stress-strain representations (Figure 1), the maximum compressive stress values and the corresponding maximum strain values were extracted (Figure 2) as an equivalent of the material’s compressive strength.

In the case of hydrogels containing only XG or PVA, a typical behavior for materials with relatively low mechanical strength was observed, independent of the cryogenic cycle (Figure 2A). In such materials, a large deformation is obtained when a low stress is applied. When switching to the XG/PVA blend composite hydrogel with the lowest content, PVA (weight ratio between XG and PVA of 9:1), the overall behavior of the material does not change significantly (Figure 2B). However, a slight tendency of decreasing the maximum deformation with increasing the corresponding maximum stress is observed with an increasing number of cycles. This effect is clearly more pronounced when the weight ratio between XG and PVA is raised to 3:1 (Figure 2C). The increase in compressive strength with the number of cycles is a process that shows how the cryogel phase progresses in the material by increasing the number of hydrogen bonding interactions. Thus, by cryogelation in successive cycles, a forced alignment of XG chains occurs due to the increase in local polymer concentration as a consequence of the ice crystal formation and decrease of water in the liquid phase. This is a mechanism by which stiff, rod-like XG chains are associated through intermolecular XG-XG hydrogen bonds [50].

By increasing the PVA content in the composite networks, such interactions are progressively disrupted. Moreover, PVA under freezing conditions leads to the formation of micro-crystallites [51], which represent cross-linking centers through hydrogen bonds, with which XG interconnects, thus forming an extensive 3D network. In this way, for the composite hydrogel with a weight ratio between XG and PVA of 3:1 prepared in seven cycles, the maximum deformation becomes considerably smaller when is compared to the maximum stress (Figure 2C). These values indicate a threshold of synthesis parameters above which the compressive strength of the material increases significantly.

Further increasing the weight ratio between XG and PVA to 1:1 led to a considerably diminished maximum deformation even after the fifth cryogenic cycle (Figure 2D). Thus, for this composition, the mechanical strength of the material increases even for the fifth cryogenic cycle, whereas for the hydrogels prepared in the III cryogenic cycles, the strength remained at the same level as for the other compositions in lower cycles. The increase in strength of the composite hydrogel CG50F is determined both by the microcrystals generated under freezing conditions by the PVA as physical cross-linking centers and by the flexibility of the PVA chain. These characteristics provide a better association through intermolecular linkages between PVA and xanthan macromolecular chains, reinforcing the 3D network specific to cryogels and leading to materials with properties different from those of the precursors. Thus, for CG50F hydrogel the best mechanical strength is obtained for cryogenic cycle VII, where the maximum applied stress relative to strain is quite high compared to the other samples. Furthermore, from the slope of the linear part of the stress−strain curves (Figure S2, supporting information), the elastic modulus (G, kPa) of the polyphenol-loaded XG/PVA composite hydrogels was determined, according to the standard procedure already established for macroporous hydrogel-based materials [33,52,53].

**Figure 3:**
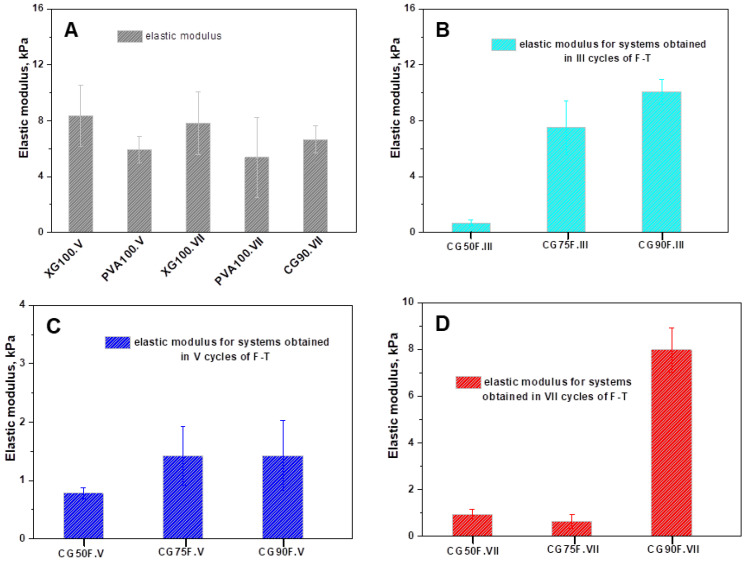
Elastic modulus of the polyphenol-loaded XG/PVA composite hydrogels: (**A**) control samples; (**B**) composite hydrogels prepared in III cycles of F-T; (**C**) composite hydrogels prepared in V cycles of F-T; (**D**) composite hydrogels prepared in VII cycles of F-TAll the data were expressed as the mean of three individual measurements ± SD.

The elastic modulus values of the polyphenol-loaded XG/PVA composite hydrogels are presented in Figure 3, in comparison with those obtained for control samples (XG100.V, XG100.VII, PVA100.V, PVA100.VII, and CG90.VII).

The elasticity of XG-based composite hydrogels was significantly affected by the PVA content, the presence of Feteasca Neagra polyphenolic extracts, and the number of cryogenic cycles (Figure 3). Tough and flexible hydrogel films were recorded for the XG-based hydrogels, with a weight ratio of 1:1 between XG and PVA and Feteasca Neagra polyphenolic extracts. The improvement of the mechanical properties of the XG-based composite hydrogels is also due to the presence of intermolecular hydrogen bonding between the functional groups of XG, PVA, and resveratrol, which reinforced the whole cryogel network. Similar results have also been reported for agar/PVA hydrogels loaded with tannic acid [54].

### 2.2. Morphological Features

**Figure 4:**
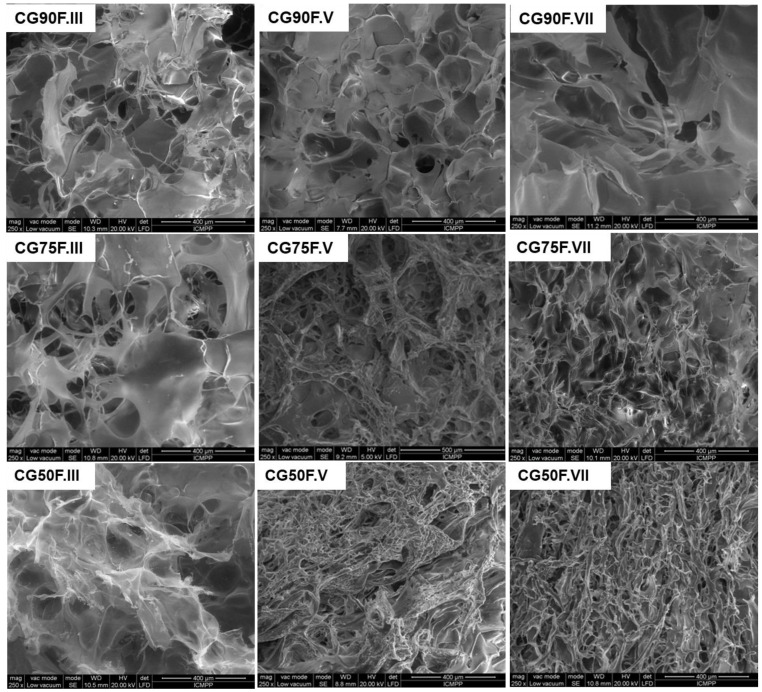
SEM micrographs of the lyophilized XG-based composite hydrogels.

In the literature, it was shown that the mechanical strength of porous materials is influenced by the pore diameters; the hydrogel networks comprised of well-connected small pores exhibited an improvement in the compressive strength [55,56]. Therefore, we analyzed our lyophilized XG-based composite hydrogels by SEM (Figure 4).

**Figure 5:**
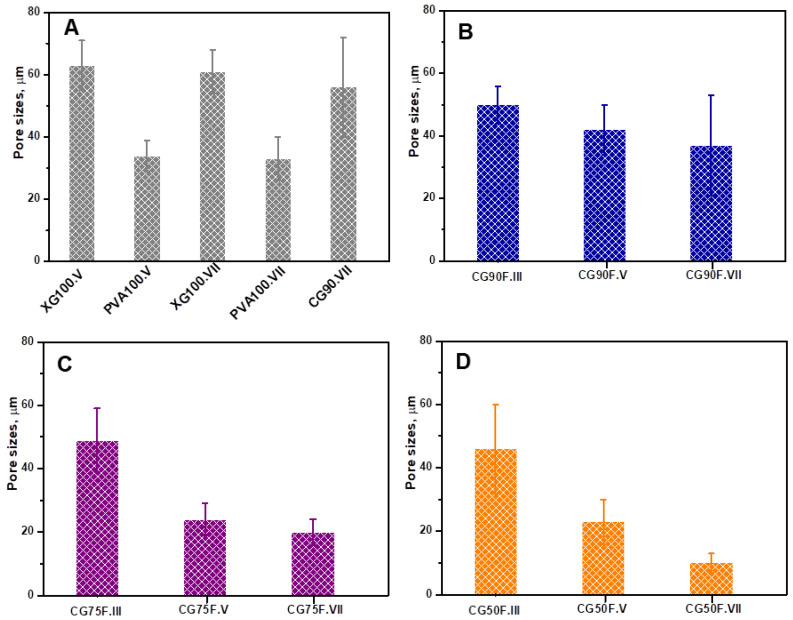
Average pore sizes of the polyphenol-loaded XG/PVA composite hydrogels: (**A**) control samples; (**B**) composite hydrogels prepared with a weight ratio between XG and PVA of 9:1; (**C**) composite hydrogels prepared with a weight ratio between XG and PVA of 3:1; (**D**) composite hydrogels prepared with a weight ratio between XG and PVA of 1:1. The pore sizes were evaluated from three individual SEM micrographs by Image J 1.48v analyzing software. All the data were expressed as the mean ± SD.

SEM micrographs of polyphenol-loaded XG/PVA composite hydrogels with weight ratios between XG and PVA of 9:1, 3:1, and 1:1 obtained by III, V, or VII cryogenic cycles show a heterogeneous morphology with interconnected pores, irrespective of network composition or synthesis conditions (Figure 4). An influence of the composition of the hydrogel network and number of cryogenic cycles is observed on the pore sizes and pore wall thickness. The mean pore sizes (Figure 5) decreased, and thinner walls were obtained by either increasing PVA content in the hydrogel matrix or by raising the number of cryogenic cycles. 

The improvement of the mechanical properties of the composite hydrogels containing a weight ratio of 1:1 between XG and PVA compared to the ones with a weight of ratio 3:1 or 9:1 could be attributed to the decrease in pore sizes and the increase in wall thickness in the former cases (Figure 4 and Figure 5). Our results are in good agreement with the data previously reported for CS/dextrin cryogels [33], CS-based polyelectrolyte complex cryogels [52], XG/Alg-based biomaterials [53], and salecan/PVA hydrogels prepared by freeze/thawing approach [57].

**Figure 6:**
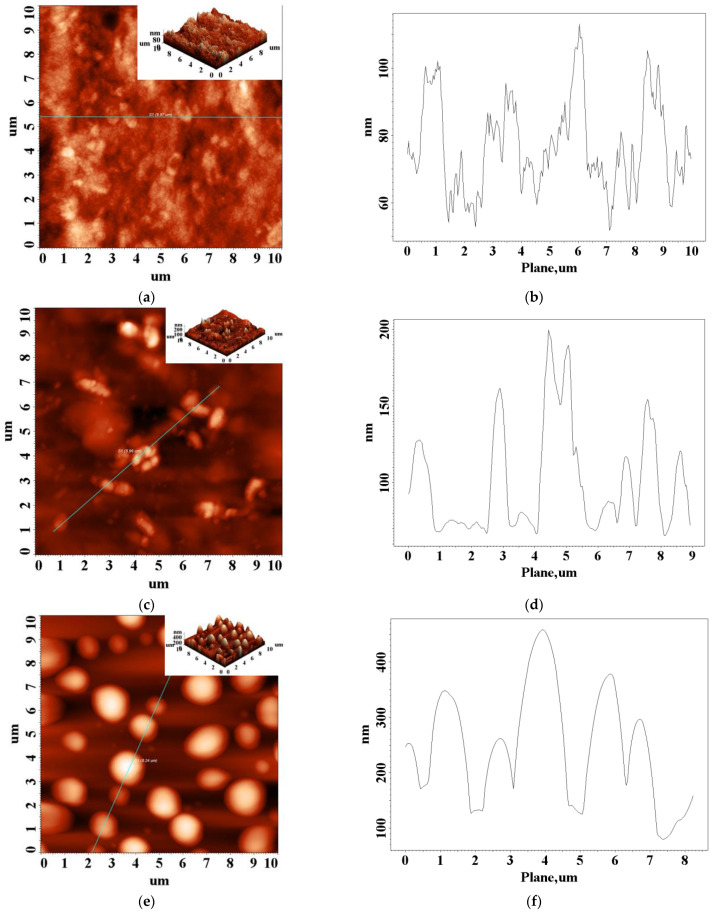
Height-contrast AFM 2D images (3D images as inset) and profiles along the straight line: (**a**,**b**): XG100.VII; (**c**,**d**): XG90PVA.III, and (**e**,**f**): XG50PVA.III.

The mechanical behavior of the XG-based composite hydrogels was also correlated with the AFM analysis. In Figure 6 are presented some 2D and 3D AFM images for XG-based composite hydrogels in comparison with those registered for XG hydrogels. In Table 2, the values obtained for the root mean square roughness at various scanning areas are presented. The images obtained by AFM scanning reveal different morphologies (Figure 6) that are specific for each network composition studied in this work.

Additionally, for the pristine XG hydrogel, the image shows a surface with random, poorly defined aggregation (Figure 6a). A ratio between XG and PVA of only 9:1 causes a considerable change in surface morphology of hydrogel. The aggregates still exist but tend to associate into new formations as small sticks that did not exist in the pure XG hydrogel films. Taking into account the low content of PVA, the new morphology obtained for XG90F.III samples indicates a specific interaction between XG and PVA that induces a new arrangement of the polymer chains in the 3D network of the network (Figure 6c). This change in the network microstructures can also be associated with the uniaxial compression data, which showed an increased rigidity for this composite hydrogel (see Figure 1, Figure 2 and Figure 3).

A totally different morphology was revealed by AFM, for the composite cryogels with a weight ratio between XG and PVA of 1:1. The XG50F.III hydrogel composite films showed very well defined structures and were considerably higher (Figure 6e). All AFM images show a progressive effect of the XG-PVA intermolecular interaction. First of all, by freezing the water solution of pure XG or an XG/PVA blend, the gelation process begins under super-cooled conditions. The progressive crystallization of water leads to the concentration of polymer chains in the unfrozen liquid micro-phase [51]. Beyond a limit of concentration, the XG polymer chains are forced to align through XG–XG interaction into a 3D network stabilized by hydrogen bonds [50]. In the XG/PVA network, this alignment can be partially interrupted by mixed XG–PVA interactions, generating a different 3D network due to the different orientation of polymer chains. In the super-cooled state of freezing water, this network experiences a micro-environmental physical stress that advances with the movement of the crystallization fronts. This can be considered a driving force that leads to the formation of specific surface morphology in the network characteristic of the polymer-polymer type interaction [51].

Adding more PVA to the system increases the number of XG–PVA intermolecular interactions at the expense of XG–XG interactions. In the composite hydrogel, with a weight ratio of 1:1 between XG and PVA, this seems to strengthen the 3D network by increasing the cross-linking density through hydrogen bonds and thus reinforcing the whole structure. As a consequence, the free energy of the surface increases and the micro-environmental physical stress increases, leading to a rougher surface (Table 2) and well-defined structures (Figure 6e).

### 2.3. Viscoelastic Properties

The connection between the viscoelastic properties of the XG-based composite hydrogels and their microstructures was evaluated by performing both steady and dynamic rheological analysis. The rheological properties of these complex gels were analyzed, taking into account the hydrogel composition (XG/PVA), the lyophilization process, and the number of freeze-thaw cycles. Moreover, we prepared systems without cryogenic freezing (sample S50F) and with the same drying history but without any freeze/defrost cycles, i.e., with zero number of cycles (sample CG50F.0) as controls.

**Figure 7:**
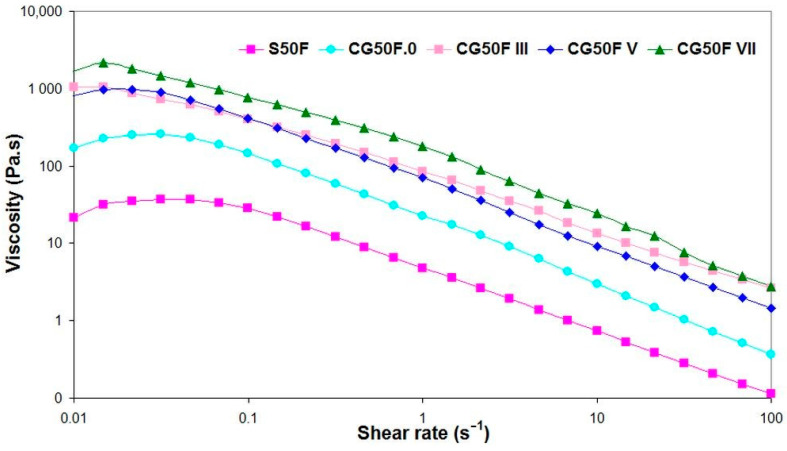
Viscosity as a function of shear rate for XG-based composite hydrogels obtained with a weight ratio between XG and PVA of 1:1, and Feteasca Neagra polyphenolic extracts in initial state, lyophilized or cryogenated at different cryogenic cycles.

The results of the rotational tests with controlled shear rate (CSR) showed different viscosity behaviors for the hydrogels in their initial state, lyophilized, and cryogenated at different cryogenic cycles (Figure 7).

The hydrogels prepared by cryogelation demonstrate higher viscosity for all shear rates tested range compared to the samples obtained in solution (S50F) or by lyophilization (CG50F.0) (Figure 7). The probable reason could be the interactions between the components induced by the freeze-thaw process that led to the formation of a stiffer hydrogel. For all samples except CG50F III, a sudden increase in viscosity is observable in the flow curves from low shear rates or approaching zero, where the materials do not flow at rest, to a maximum value where one could mention that the hydrogels tend to resist flow [58,59]. A very small Newtonian region corresponding to the steady plateau at low values of shear rates, when almost no change in viscosity could be observed in the steady-state flow curve of S50F, decreasing for CG50F.0 (Figure 7), was followed by a non-Newtonian behavior at shear rates higher than 0.1 s^−1^. By increasing the shear rate, all hydrogels flow under the applied deformation, indicating shear thinning of a non-Newtonian nature. The composite cryogel obtained with the highest number of cryogenic cycles is the stiffer material among all CG50F hydrogels.

**Figure 8:**
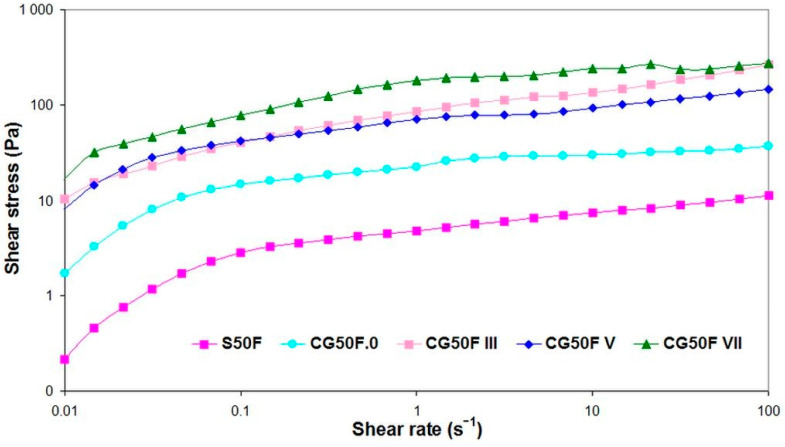
Rheograms (shear stress vs. shear rate) recorded for XG-based composite hydrogels obtained with a weight ratio between XG and PVA of 1:1, and Feteasca Neagra polyphenolic extracts in initial state, lyophilized and cryogenated at different cryogenic cycles.

The shear stress against shear rate rheograms for the studied hydrogels displayed in Figure 8 show an increasing trend of the shear stress with increasing shear rate, which confirms the shear thinning non-Newtonian behavior of the hydrogels.

**Figure 9:**
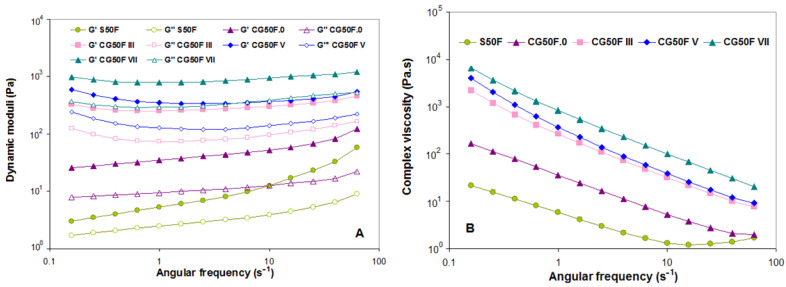
Effect of the lyophilization and cryogenic cycles number (III, V and VII) on the variation of (**A**) storage modulus (G′) (solid symbols) and loss modulus (G″) (open symbols) and (**B**) complex dynamic viscosity (|η*|) function of angular oscillation frequency at 25 °C for XG-based composite hydrogels obtained with a weight ratio between XG and PVA of 1:1, and Feteasca Neagra polyphenolic extracts.

The results obtained by performing frequency sweep tests (Figure 9) indicate that cryogelation led to stiffer hydrogels (higher G′ and G″ values) than those initially formed (without any further processing) or lyophilized hydrogels (CG50F.0).

We note that, for the samples S50F and CG50F.0, both dynamic moduli are slightly dependent on the frequency in our experimental range, more obvious for initially formed hydrogel at higher frequencies. The shapes of these curves are similar to those of soft materials with a gel-like structure [60,61]. The increase of both G′ and G″ as well as of complex viscosity values for cryogenized hydrogels compared to the initial and lyophilized hydrogels shows that the additional cryogenic cycles resulted in significant mechanical stiffness based on a strengthening of the gel network under these latter conditions, resulting in hydrogels more resistant to deformation with gel-like behavior (G′ > G″).

**Figure 10:**
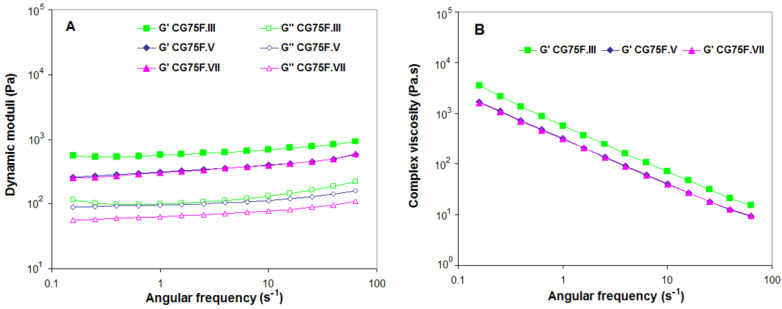
Effect of the cryogenic cycles number (III, V and VII) on the variation of (**A**) storage modulus (G′) (*solid symbols*) and loss modulus (G″) (*open symbols*), (**B**) complex dynamic viscosity (|η*|) function of angular oscillation frequency at 25 °C for XG-based composite hydrogels obtained with a weight ratio between XG and PVA of 3:1, and Feteasca Neagra polyphenolic extracts.

**Figure 11:**
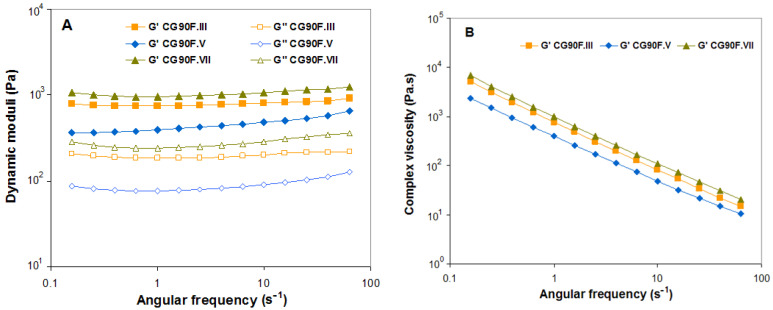
Effect of the cryogenic cycles number (III, V and VII) on the variation of (**A**) storage modulus (G′) (*solid symbols*) and loss modulus (G″) (*open symbols*), (**B**) complex dynamic viscosity (|η*|) function of angular oscillation frequency at 25 °C for XG-based composite hydrogels obtained with a weight ratio between XG and PVA of 9:1, and Feteasca Neagra polyphenolic extracts.

The angular frequency (ω) dependence of storage modulus (G′) and loss modulus (G″) as well as of the complex dynamic viscosity (η*), for the XG-based composite hydrogels obtained with a weight ratio between XG and PVA of 3:1 or 9:1, and Feteasca Neagra polyphenolic extracts, are plotted in Figure 10 and Figure 11.

The gel behavior is confirmed for all evaluated cryogels, as the elastic modulus (G′) is significantly greater than the viscous modulus (G″) for the entire frequency range [42,62,63] (Figure 9A, Figure 10A and Figure 11A). A decreasing trend of the complex viscosity was recorded with increasing frequency (Figure 9B, Figure 10B, and Figure 11B).

The polyphenol-laden composite hydrogel with a weight ratio of 3:1 between XG and PVA obtained by III cryogelation cycles displayed an increase in hydrogel stiffness. This behavior was also observed in the uniaxial compression tests (see Section 2.1) and was associated with the changes in XG-based composite hydrogel microstructures (see SEM micrographs in Figure 4 and AFM images in Figure 6) as a consequence of freeze-thawing conditions. In the case of the CG75F composite hydrogels prepared by V or VII freeze-thawing cycles, the elastic behavior of cryogels is almost similar, with both G’ curves almost superimposing and an identical trend for complex viscosity over all ω tested range. For the composite hydrogels with a weight ratio between XG and PVA of 1:1 synthesized by III or V freeze-thawing cycles, both storage and loss moduli were found to be most dependent on angular frequency, indicating more soft materials at this composition. On the other hand, the dynamic moduli were almost independent for the stiffer hydrogel composite obtained after VII freeze-thawing cycles (sample CG50F.VII), having a slightly soft consistency, especially for ω between 1 and 10 s^−1^. The XG-based composite hydrogels with a weight ratio of 1:1 between XG and PVA obtained in III or V cryogenic cycles (samples CG50F.III and CGF50.V) showed nearly similar elastic (G′) and flow (η*) behavior at high oscillation frequencies. These results are in agreement with the data obtained for the uniaxial compression measurements (see Section 2.1). As Figure 9 shows, the increasing of cryogenic cycles from III to VII led to the enhancement of the dynamic moduli (G′ and G″) only for the composite hydrogels obtained with a weight ratio of 1:1 between XG and PVA. Fernandes et al. [37] showed that the helical structure of linear arabinans favors the formation of strong associations with polyphenols due to the appearance of hydrophobic domains by “polymer entanglement.” Moreover, the interaction of polysaccharides with hydrophobic polyphenols “by stacking” gives the possibility of their self-association by hydrophobic interactions [37]. A similar helix structure occurs in XG under cryogenic conditions when the side chains are packed around the main chain. By introducing PVA into the polymer blend, the helix structure of XG is perturbed due to heteropolymeric interactions. In this case, the hydrophobic domains are reduced, and the interaction between polyphenols and polymers is oriented towards hydrogen bonds, reinforcing the polymer matrix and thus enhancing their mechanical properties.

A power law frequency dependence of G′, G″, and η* was registered for most of the samples, except for those prepared with a weight ratio between XG and PVA of 1:1, which can be approximated with the simple models from Equations (1)–(3) [64,65,66]. Similar behavior has been observed by Tao et al. for hydrogels based on XG cross-linked with sodium trimetaphosphate [67] and Popescu et al. for alginate/poloxamer hydrogels [68].

G′(ω) = G_0′_ ω ^n′^, (1)

G″(ω)~ω ^n″^ (2)

η* (ω)~ω ^m^ (3)

where G_0′_ refers to the gel stiffness, representing the energy stored and recovered per cycle of sinusoidal shear deformation, and ω is the oscillation frequency.

Increased values of G_0′_ indicate the rigidity of the materials, which is associated with the formation of an elastic gel structure [69].

The power law parameters resulting from frequency sweep tests are displayed in Table 3 as a function of the hydrogel composition and freeze-thawing cycles.

The increase in PVA amount in the hydrogel network led to a decrease in gel stiffness and viscoelasticity, respectively (see G_0′_ values in Table 3) that could be related to the hydrogel formation mechanism. PVA is associated with the generation of an increase number of hydrogen bonds, the higher PVA content, the longer is the time needed to promote hydrogel formation [70].

The increase of G_0′_ values indicates an enhancement of the sample’s stiffness, most evident for CG75F.III, while the n′ and n″ exponent values impart information on the variation of the dynamic moduli with angular frequency. The relative low values of n′ and n″ indicate that G′ and G″ are almost independent on frequency. Decreased n′ and n″ parameters are characteristics of less frequency-dependent, stronger hydrogels [71].

The values of m, which is the complex viscosity relaxation exponent of a hydrogel network, are related to liquid-like behavior when they are close to zero and a solid-like response when they approach −1 [70]. In the case of our developed cryogels, the m values vary between −0.8806 for CG75F.VII and −0.9744 for CG90F.III, with a tendency to more negative values for higher XG.

## 3. Conclusions

In this study, we successfully investigated the compressive and viscoelastic properties of some physically cross-linked xanthan gum/poly(vinyl alcohol) (XG/PVA) composite hydrogels containing polyphenolic extracts derived from Feteasca Neagra red grape pomace. It was found that the mechanical parameters of the XG-based composite hydrogels, such as elasticity, stiffness, compressive strength, and deformation strain, can be controlled by the number of cryogenic cycles, ratio between polymeric components, and the incorporation of bioactive compounds.

Tough and flexible polyphenol-loaded composite films were obtained for the hydrogel with a weight ratio of 1:1 between XG and PVA. The SEM analysis revealed a heterogeneous morphology with interconnected pores for all composite hydrogels. It was shown that the mean pore sizes decreased and thinner walls were obtained by either increasing PVA content in the hydrogel matrix or by raising the number of cryogenic cycles. These results were correlated with the improvement of the mechanical properties of the composite hydrogels with a weight ratio of 1:1 between XG and PVA, compared to the ones with a weight ratio of 3:1 or 9:1.

The AFM data further supported the uniaxial compression and rheological results by revealing the fine microstructure of the composite hydrogel films, which was changed by increasing the PVA content in the hydrogel networks.

The gel behavior was confirmed for all composite hydrogels by oscillatory shear measurements under small deformation conditions. The physical cross-linking of natural polymers to obtain various materials has the advantage of avoiding secondary reaction products or unreacted reagents.

The aim of this manuscript was to investigate, as a proof-of-concept, the role of the synthesis parameters and the incorporation of natural polyphenolic extract within hydrogel networks on the mechanical and morphological properties of physically cross-linked XG/PVA composite hydrogels. Consequently, further work is still needed to optimize the ratio between the used materials and their resultant properties before considering their potential application in food packaging.

## 4. Materials and Methods

### 4.1. Materials

Three-dimensional composite hydrogels consisting of XG, PVA, and Feteasca Neagra grape pomace extracts were investigated in this study by oscillatory shear measurements and uniaxial compression tests. These materials were prepared by multiple cryogenic cycles combined with lyophilization as a method of drying, according to a procedure previously described [13]. The raw materials, i.e., XG and PVA (99% hydrolyzed), used for composite hydrogel preparation, were purchased from Sigma-Aldrich (St. Louis, MO, USA) and used as received. The viscometric average molecular weight (M_v_) of XG was 1.98 × 10^6^ g mol^−1^. The number-average molar mass (M_n_) and polydispersity index (PDI) of PVA were determined by gel permeation chromatography (GPC) as 97 × 10^3^ g mol^−1^ and 1.17, respectively. The polyphenolic extracts derived from the Feteasca Neagra type grape pomace entrapped within our hydrogel films were provided by the “Ion Ionescu de la Brad” University of Agricultural Sciences and Veterinary Medicine. The chemical composition of the Feteasca Neagra type grape pomace sample had already been evaluated by high performance liquid chromatography (HPLC) [72,73], which indicated trans-resveratrol as the main compound in our sample. By Folin–Ciocalteu method was determined a total phenolic content in the Feteasca Neagra type grape pomace extract of about 1.452 mg gallic acid/mL.

### 4.2. Methods

#### 4.2.1. Water Swelling Measurements

The swelling behavior of polyphenol-loaded XG/PVA composite hydrogels in distilled water was gravimetrically investigated by soaking a defined amount of dried film (0.01 g) in 10 mL aqueous solutions. The swelling ratio (*SR*, g/g) was calculated as:

(4) $$SR=\frac{W_{t}}{W_{d}},$$

where *W_t_* and *W_d_* are the weights of swollen films at time *t* and, respectively, dried films. All the data were expressed as the mean of three individual measurements ± SD.

#### 4.2.2. Water Contact Angle Evaluation

The wettability of polyphenol-loaded XG/PVA composite hydrogel films was evaluated by performing static contact angle (θ°) tests using the drop method, as previously applied for XG/lignin hydrogel films [12]. The θ° values were determined by fitting the Young–Laplace equation and are reported in Table 1 as the means of six successive measurements. All the data were expressed as the mean of five individual tests ± SD.

#### 4.2.3. Rheological Measurements

The rheological characterization of the polyphenol-loaded XG/PVA composite hydrogels in a swollen state was performed by means of a controlled stress rheometer, Physica MCR 301 (Anton Paar, Graz, Austria), using parallel-plate geometry with an upper diameter of 25 mm. Steady shear and dynamic studies were realized at 25 ± 0.1 °C. The samples were transferred in a dried state to the center of the lower plate of the rheometer, followed by their hydration with MilliQ water before the initiation of the rheological measurements. The temperature and evaporation were controlled by a Peltier system; therefore, constant sample hydration was maintained during testing. The upper plate was lowered until it reached the sample surface, the excess hydrogel being removed so that the hydrated material correctly filled the geometry; a fixed gap of 1 mm was used for measurements. The flow behavior of the XG-based composite hydrogels obtained with a weight ratio of 1:1 between XG and PVA and Feteasca Neagra polyphenolic extracts was assessed by a controlled shear rate (CSR) test, where the dependence of the shear stress and viscosity on shear rate was followed. The viscoelastic properties of the hydrogels were evaluated by oscillatory frequency sweep tests realized in the linear viscoelastic region (LVE) with a previously determined fixed strain of 5% in the frequency domain between 0.05 and 500 rad/s. The performed rheological measurements by means of the stress-controlled rheometer MCR 301 (Anton Paar, Austria) and are highly precise due to the error-free Toolmaster™ technology, which automatically recognizes the measuring systems and transfers all relevant parameters to the software without the risk of errors, and the TruGap™ system, which offers permanent control of the measuring gap. Upon three consecutive measurements on different samples of the same cryogel, it has been noted that the standard deviations were up to ~2% for the recorded rheological data.

#### 4.2.4. Uniaxial Compression Tests

The mechanical tests were carried out on lyophilized polyphenol-loaded XG/PVA composite hydrogels. Thus, plate-shaped samples with a thickness, width, and height of about 8 mm, 6 mm, and 4 mm were uniaxially compressed using a Shimadzu Testing Machine (EZ-LX/EZ-SX Series, Kyoto, Japan). A cross-head speed of 0.2 mm min^−1^ and a force of 20 N were used to analyze all samples. The strain (ε), the compressive nominal stress (σ, kPa), and the elastic moduli (G, kPa) of all samples were evaluated taking into account the previously published procedure [74]. All the data were expressed as the mean of three individual tests ± SD.

#### 4.2.5. Scanning Electron Microscopy (SEM)

A Quanta 200-FEI-type environmental scanning electron microscope (ESEM), operating at 20 kV in low vacuum mode, was used to analyze the cross-sectional microstructure of the lyophilized polyphenol-loaded XG/PVA composite hydrogels. Image J 1.48v analyzing software was involved to determine the mean values of the pore sizes of all samples [75]. The mean values ± SD were obtained by measuring at least 30 pores (voids) for each sample.

#### 4.2.6. Atomic Force Microscopy (AFM)

A NTEGRA scanning probe microscope (NT-MDT Spectrum Instruments, Moscow, Russia), in atomic force microscopy (AFM) configuration, was used to collect the 2D and 3D surface images for the polyphenol-loaded XG/PVA composite hydrogel films. The film surfaces were scanned using rectangular silicon cantilevers NSG 03 (NT-MDT, Russia) having tips of equal height and aspect ratio. The Nova v.19891 for Solver software was used to process the AFM images and calculate the surface parameters [76].

## Figures and Tables

**Table 1:** Sample codes, composition, and some characteristics of composite hydrogels.

Sample Code	XG:PVA	Amount of Extract, *v*/*v*%	Swelling Ratio ^1^ g/g	Contact Angle ^2^ θ°
XG100.V	-	0	63 ± 7	70 ± 3
PVA100.V	-	0	-	89 ± 4
XG100.VII	-	0	62 ± 10	71 ± 4
PVA100.VII	-	0	-	88 ± 4
CG90.VII	9:1	0	59 ± 9	79 ± 3
CG90F.III	9:1	10	22 ± 8	81 ± 5
CG90F.V	9:1	10	20 ± 5	82 ± 6
CG90F.VII	9:1	10	20 ± 3	83 ± 4
CG75F.III	3:1	10	21 ± 3	81 ± 4
CG75F.V	3:1	10	17 ± 5	85 ± 3
CG75F.VII	3:1	10	16 ± 6	85 ± 4
CG50F.III	1:1	10	13 ± 5	83 ± 3
CG50F.V	1:1	10	12 ± 5	87 ± 3
CG50F.VII	1:1	10	11 ± 3	92 ± 6

^1^ The swelling ratio was determined in distilled water by a gravimetric method and calculated with Equation (4) (see Section 4. Materials and Methods); ^2^ Contact angle (θ°) values were estimated by fitting the Young–Laplace equation (see Section 4. Materials and Methods).

**Table 2:** Values of the root mean square roughness for XG-based composite hydrogels.

Scanning Area ^1^	5 × 5 μm^2^	10 × 10 μm^2^	20 × 20 μm^2^	30 × 30 μm^2^	40 × 40 μm^2^
XGVII	17 ± 0.2	18 ± 1	22 ± 8	42 ± 2	63 ± 1
XG90F.III	23 ± 1	25 ± 1	30 ± 4	40 ± 3	46 ± 5
XG50F.III	76 ± 12	85 ± 5	92 ± 2	95 ± 3	98 ± 1

^1^ All the data were expressed as the mean ± standard deviation (SD).

**Table 3:** The experimental results of n′, n″, m, A, and R^2^ values of XG-based composite hydrogels.

Sample	G_0′_ (Pa)	n′	n″	−m	R^2^ (G′)	R^2^ (|η*|)
CG90F.III	766 ± 15	0.026	0.020	−0.974	0.652	0.999
CG90F.V	399 ± 8	0.093	0.066	−0.907	0.944	0.999
CG90F.VII	1003 ± 21	0.033	0.056	−0.965	0.574	0.999
CG75F.III	582 ± 10	0.087	0.115	−0.912	0.912	0.999
CG75F.V	313 ± 5	0.121	0.085	−0.881	0.968	0.999
CG75F.VII	305 ± 4	0.130	0.100	−0.871	0.967	0.999

## Data Availability

Not applicable.

## Supplementary Materials

The following supporting information can be downloaded at: https://www.mdpi.com/article/10.3390/gels9040281/s1, Figure S1: Linear dependence of the stress-strain data for XG-based composite hydrogels; Figure S2. Linear dependence of the stress-strain data for XG-based composite hydrogels.
