# Supplementary material for: Correlation between Mechanical and Morphological Properties of Polyphenol-Laden Xanthan Gum/Poly(vinyl alcohol) Composite Cryogels
Source: Gels. 2023 Mar 29;9(4):281. doi: 10.3390/gels9040281 (PMC10137999; doi:10.3390/gels9040281)
Supplement: Supplementary file 1 [file gels-09-00281-s001.zip › gels-2321176-supplementary.pdf]

Supplementary Materials:

Correlation between Mechanical and Morphological Properties of Polyphenol-Laden Xanthan Gum/Poly(Vinyl Alcohol) Composite Cryogels

Irina Elena Raschip *, Raluca Nicoleta Darie-Nita, Nicusor Fifere, Gabriela-Elena Hitruc, Maria Valentina Dinu

Petru Poni" Institute of Macromolecular Chemistry, Grigore Ghica Voda Alley 41A, 700487 Iasi, Romania

* Correspondence: iecoj@icmpp.ro (I.E. Raschip)

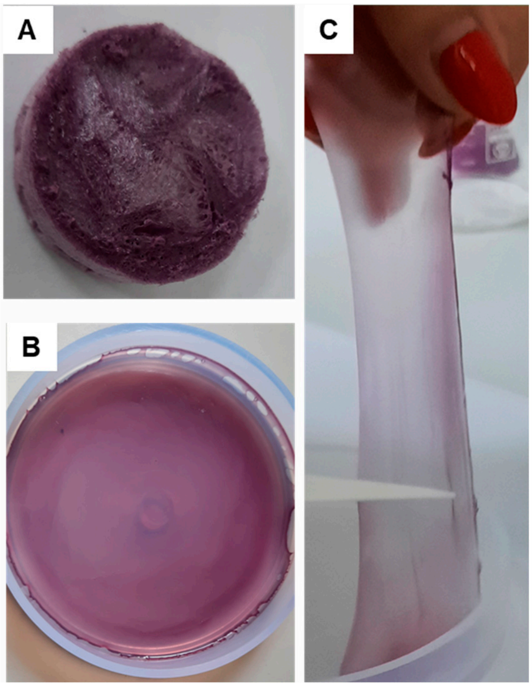

Figure S1. Optical images of the polyphenol-loaded XG/PVA composite hydrogels prepared with a weight ratio between XG and PVA of 1:1, and 10 v/v% Feteasca Neagra polyphenolic extracts dried by lyophilization (A) and solvent casting (B, C).

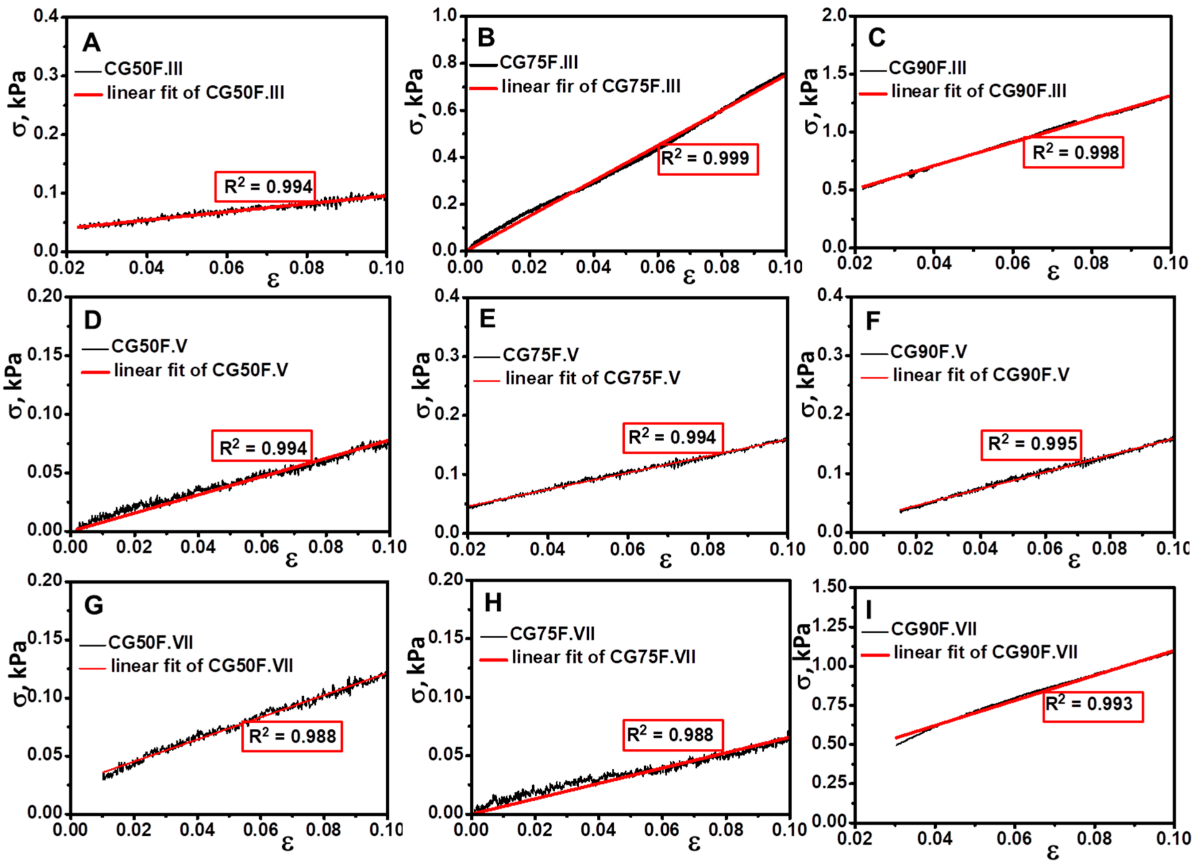

Figure S2. Linear dependence of the stress-strain data for XG-based composite hydrogels.
